# Mesenchymal Stromal Cell Therapy in Ischemia/Reperfusion Injury

**DOI:** 10.1155/2015/602597

**Published:** 2015-07-15

**Authors:** Pascal Rowart, Pauline Erpicum, Olivier Detry, Laurent Weekers, Céline Grégoire, Chantal Lechanteur, Alexandra Briquet, Yves Beguin, Jean-Marie Krzesinski, François Jouret

**Affiliations:** ^1^Division of Nephrology and Transplantation, University of Liège CHU (ULg CHU), 4000 Liège, Belgium; ^2^Laboratory of Cardiovascular Sciences, Groupe Interdisciplinaire de Génoprotéomique Appliquée (GIGA), University of Liège, 4000 Liège, Belgium; ^3^Division of Abdominal Surgery and Transplantation, University of Liège CHU (ULg CHU), 4000 Liège, Belgium; ^4^Laboratory of Hematology, Groupe Interdisciplinaire de Génoprotéomique Appliquée (GIGA), University of Liège, 4000 Liège, Belgium; ^5^Laboratory of Cell and Gene Therapy, University of Liège CHU (ULg CHU), 4000 Liège, Belgium

## Abstract

Ischemia/reperfusion injury (IRI) represents a worldwide public health issue of increasing incidence. IRI may virtually affect all organs and tissues and is associated with significant morbidity and mortality. Particularly, the duration of blood supply deprivation has been recognized as a critical factor in stroke, hemorrhagic shock, or myocardial infarction, as well as in solid organ transplantation (SOT). Pathophysiologically, IRI causes multiple cellular and tissular metabolic and architectural changes. Furthermore, the reperfusion of ischemic tissues induces both local and systemic inflammation. In the particular field of SOT, IRI is an unavoidable event, which conditions both short- and long-term outcomes of graft function and survival. Clinically, the treatment of patients with IRI mostly relies on supportive maneuvers since no specific target-oriented therapy has been validated thus far. In the present review, we summarize the current literature on mesenchymal stromal cells (MSC) and their potential use as cell therapy in IRI. MSC have demonstrated immunomodulatory, anti-inflammatory, and tissue repair properties in rodent studies and in preliminary clinical trials, which may open novel avenues in the management of IRI and SOT.

## 1. Introduction

Ischemic injury occurs when the blood supply to a tissue or an organ is stopped. The consequences of depriving an organ of its blood supply have long been recognized as a critical factor in the clinical outcomes of stroke, hemorrhagic shock, and myocardial infarction, as well as in solid organ transplantation (SOT). The incidence of ischemic injury events affects more than 1.3 million individuals each year in USA alone. Prolonged ischemia results in multiple cellular metabolic and ultrastructural changes. It may cause, among others, deprivation of oxygen leading to a fall of ATP and the upregulation of glycolysis to avoid such a decrease. The upregulation of glycolysis leads to subsequent production of lactic acid and intracellular acidosis. Ischemia can also alter membrane potential, ion transporter distribution, and cytoskeletal disorganization [1]. Following the ischemic insult, the reperfusion of damaged tissues induces both local and systemic inflammation. Tissular and cellular damage after reperfusion of previously viable ischemic tissues is defined as ischemia/reperfusion injury (IRI). IRI causes widespread microvascular dysfunction and altered tissue barrier function. If severe enough, the inflammatory response after IRI may even induce a systemic inflammatory response or multiple organ dysfunction syndromes, which account for up to 30–40% of intensive care unit mortality. In the particular field of SOT, IRI is unavoidable. Although IRI-associated damage can be attenuated by storing the organ in a cold solution (“cold ischemia”), it cannot be completely prevented. Still, IRI may be responsible for delayed graft function (DGF), with short- and long-term consequences on organ function and survival [2].

In this review, we summarize the current literature on mesenchymal stromal cells (MSC) and their potential use as cell therapy in cases of ischemia. Indeed, MSC have demonstrated immunomodulatory and tissue repair properties in rodent studies and in preliminary clinical trials, which may open novel avenues in the management of IRI and SOT.

## 2. Properties of Mesenchymal Stromal Cells

MSC represent a heterogeneous population of adult fibroblast-like multipotent cells which can differentiate themselves into various mesodermal lineages. MSC can be found in many tissues, including bone marrow, umbilical cord, muscle, or adipose tissue [3]. MSC have been defined by the International Society for Stem Cell Research as plastic adherent cells, with an attached fibroblast-like morphology in standard conditions, which can be differentiated into adipocytes, chondrocytes, and osteoblasts under standard* in vitro* differentiating conditions. In addition, they must express the mesenchymal markers CD105, CD90, and CD73 but importantly not express the haematopoietic markers CD45, CD34, CD14, CD79a, CD11b, and HLA-DR [4]. MSC express few HLA class I and no HLA class II molecules, allowing them to evade allogeneic immune response. This is the so-called “immunoprivilege,” an interesting feature in MSC biology, which makes these cells extremely suitable for both autologous and allogeneic transplantation [5].

Many studies have demonstrated the immunomodulatory role of MSC, including their anti-inflammatory properties on both the innate and adaptive immune system. Indeed, MSC can exert profound immunosuppression both* in vitro* and* in vivo* by inhibiting the proliferation and function of a number of immune cell types, including T-lymphocytes, natural killer (NK) cells, and dendritic cell (DCs) [6]. In addition, MSC have been reported to prompt T cell expansion towards a regulatory phenotype. These regulatory T cells (Treg), including the naturally occurring CD25^+^FoxP3^+^ Treg in the thymus and the adaptive Treg in periphery, are responsible for maintaining tolerance to self-antigens and controlling excessive immune response to external antigens [7]. The potential mechanisms of MSC-induced Treg differentiation may involve (i) direct cell-cell contacts, (ii) the production of prostaglandin E2 and transforming growth factor *β*-1 (TGF-*β*-1), and (iii) the release of a nonclassical HLA class I molecule, HLA-G5 (Table 1) [8]. Furthermore, MSC can secrete microvesicles (MVs) and may help transfer cellular materials to neighbouring cells [9, 10]. MVs contribute to the paracrine action of MSC as integral component of the cell-to-cell communication network. They horizontally transfer mRNA, microRNA, proteins, and organelles, which may lead to functional and phenotypic changes [11]. Interestingly, various* in vitro* observations suggest that the culture conditions, the types and concentrations of cytokines in the milieu, and the activation status of T cells at the time of exposure to MSC also influence their final differentiation [12]. An* in vitro* study shows that the production of proinflammatory Th1-type cytokines, including IL-2 and IFN-g, was significantly decreased in MSC-treated rats. In contrast, the concentrations of the Th2-type cytokine IL-4 were markedly increased (Table 1) [13]. In addition to their impact on T-cell fate, the injection of MSC can also influence the macrophage outcomes. Naturally, without the intervention of MSC, the M1 macrophage phenotype is the dominant population with a proinflammatory effect by the secretion of tumor necrosis factor- (TNF-) *α* and interleukin- (IL-) 1*β* [14]. After the injection of MSC, the ratio M1/M2 changes with again a preferential shift towards an anti-inflammatory immunosuppressive M2 phenotype with the secretion of IL-10, -11, -12, and -13 (Table 1) [15, 16]. M2 macrophages have been implicated in the generation and maintenance of Treg. Finally, MSC treatment* in vitro* inhibits antigen presenting cells (APC), which further favors Treg expansion through the release of TGF-*β* [17].* In vivo*, the beneficial MSC-induced polarization of T cells toward a Treg phenotype has been demonstrated in numerous experimental models of autoimmune and inflammatory diseases, such as systemic lupus erythematosus, fibrillin-mutated systemic sclerosis, or colitis.

In addition to these immunoregulatory properties, MSC exert tissue repair functions in damaged organs [18]. In particular, experimental observations have demonstrated their protective effect in acute kidney injury (AKI), acute myocardial infarction (AMI), and liver and lungs injury [18]. Following IRI, MSC reduce inflammation and accelerate vascular supply [19]. Indeed, single or repeated injections of MSC or MSC-derived microvesicles after injury accelerate functional recovery of the kidneys [18] or the heart and improve survival in a lethal model of AKI [12]. MSC activate endogenous cellular repair programs by releasing various growth factors such as fibroblast growth factor (FGF), keratinocyte growth factor (KGF), erythropoietin (EPO), epidermal growth factor (EGF), insulin-like growth factor (IGF), keratinocyte growth factor (KGF), monocyte chemoattractant protein-1 (MCP-1), and stromal cell-derived factor-1 (SDF-1) [20]. The role of MSC in the mechanisms of angiogenesis and vascular remodelling may involve the upregulation of prosurvival and proangiogenic factors such as vascular endothelial growth factor (VEGF-a), angiopoietins (ANGPT), IGF-1, and hepatocyte growth factor (HGF). Additional MSC-related mediators, including IL-10, IL-6, TGF-*β*, or nitric oxide (NO), may further facilitate a local anti-inflammatory state, thereby allowing the healing of damaged tissues (Table 1). Recent studies also suggest that extracellular vesicles may participate in the paracrine/endocrine network involved in the MSC biologic action. Extracellular vesicles released by MSC after receptor/ligand interactions are internalized in target cells, thereby transferring proteins, bioactive lipids, and surface receptors [21]. However, it is widely accepted that the beneficial effect of BM-derived stromal cells in AKI or AMI is due to the generation of an environment that favors the proliferation of dedifferentiated epithelial cells surviving the injury rather than direct transdifferentiation of stromal cells into mature tissues [22]. Moreover, the expansion of surviving renal tubular cells observed with the administration MSC or MSC derivatives results from the induction of prosurvival genes and downregulation of proapoptotic genes. Finally, MSC-derived microvesicles help rapidly restore ATP supply following IRI by transferring mitochondria into the damaged cells [9, 10]. All these* in vitro* and* in vivo* studies show the potential effect of MSC in modulating the immunity and the reparation of different tissue and their anti-inflammatory feature via both direct cell-cell interactions and the release of paracrine factors.

## 3. Pathophysiology of Ischemia/Reperfusion Injury

A sudden and prolonged interruption of arterial blood flow with immediate oxygen and nutriment deprivation to the cells (i.e., hypoxia with accumulation of metabolic products) is defined as ischemic injury [23]. After this interruption of flow during ischemia, the reperfusion also alters the vessel by the increase of the blood flow and the sudden increasing of the oxygen concentration, resulting in the development of oxidative reactions. Molecular and biochemical changes in the vascular wall are characteristic of an acute inflammatory response. Indeed, the endothelial cells appear to be particularly vulnerable to the deleterious effects of both hypoxia (ischaemia) and reoxygenation (reperfusion). Maintained hypoxia alters membrane potential, disturbs the distribution of ions, and increases intracellular volume. Some glycolytic enzymes will be activated by the oxidative environment. The toxicity of metabolic products, which are not washed out or eliminated, increases in parallel with the osmolar load. Oedema results in disruption of cellular membranes, not only the outer cellular membrane by opening of stretch activated channels that counteract the volume increase, with dissipation of the semiconductance of the membrane, but also endoplasmic reticulum (ER), Golgi apparatus, mitochondrial membranes, and cytoskeletal microtubules [23]. Into the cells, ischemia will cause a rapid depletion of energy supplies of cells since oxidative phosphorylation can no longer proceed in the mitochondria in the absence of oxygen. Moreover, IRI is associated with a massive and local production of reactive oxygen species (ROS), which are responsible for the detrimental oxidation of proteins, lipids, membranes, and nucleic acids of both epithelial and endothelial cells [24–26].

In addition to these metabolic problems, IRI is associated with a large inflammatory response with the upregulation of the expression and activation of endothelial adhesion molecules, integrins, and selectins. This inflammation further exacerbates the injury. The inflammation occurring in IRI is called “sterile inflammation” or “damage-associated molecular pattern” (DAMP) to differentiate it from the inflammatory response to infections. Indeed, in IRI, molecules normally residing within cells elicit inflammation when they are released into the extracellular space or are expressed on cell surfaces. In addition, enzymes released by injured cells or leukocytes convert extracellular matrix molecules to proinflammatory signals. Finally, intracellular stress may generate proinflammatory signals [27]. DAMP activate the innate immune responses via the Toll-like receptors (TLRs), especially the TLR-4, and recruit inflammatory cells [28]. The deleterious impact of IRI-associated inflammation and infiltration of monocytes involves chemokine receptors, such as chemokine receptor-2, chemokine receptor-7, and CXC chemokine receptor-4, as well as the local production of ROS, TNF-*α*, and interleukin-1*β* [29]. In addition, there is a sustained amplification of IgG1 antibodies directed against an antigen encountered in the days following IRI [30]. In rodent models of renal IRI, the total amount of antigen-unspecific IgG1 and the number of B lymphocytes remain unchanged during this period, but the number of antigen-specific lymphocytes increases. This effect is lost in mice deficient in complement factor B that lack a functional alternative pathway of complement, as well as in IL10-deficient mice. These observations suggest that kidney IRI leads to a rise in antibody production against heterologous antigens [30]. Interestingly, the total amount of antigen-unspecific IgG1 and the number of B lymphocytes remain unchanged during this period, but the number of antigen-specific lymphocytes increases [30]. The role of B lymphocytes at the time of IRI remains unclear, with conflicting observations as to whether these cells are protective or harmful [31].

All these inflammatory and immune consequences may play an even more important role in IRI at the time of SOT, as detailed infra. A better understanding of the tissular and cellular phenomena associated with renal IRI would thus help exploit them to prevent or attenuate the ischemic damage [32]. Clinical research using MSC is steadily increasing, as illustrated by the number of hits found on the website https://clinicaltrials.gov/. As of March 2015, 94 trials have been registered, including 14 in IRI attenuation.

## 4. MSC Therapy in Renal Ischemia/Reperfusion Injury

In renal IRI, MSC are thought to operate through intermediate effectors involved in 2 systems: (i) the cytokine network that regulates the immune response in acute rejection and (ii) the systems that have been shown to promote repair and to modulate immune cell traffic in renal tissue in different models of kidney disease.

For the past several years, many studies have showed that MSC proved their ability to protect against IRI-associated AKI. Some of them clearly demonstrated that MSC therapy affords significant renoprotection in rats. Animals infused with MSC either immediately or 24 hours or 1 week after reperfusion had significantly better renal function, lower renal injury and apoptotic scores, and higher mitogenic indices than vehicle-treated animals [33, 34]. Twenty-four hours after their injection, not any or only exceptionally numbers of MSCs were found in the kidney. From these observations, they first deduced that the mechanisms that mediate the protective effects of MSC must be primarily paracrine, as implied by their expression of several growth factors such as HGF, VEGF, and IGF-I, all known to improve renal function in case of IRI. MSC-injected rats had significant downregulation of IFN-*γ* and simultaneous rise in IL-10 levels. These results suggest that MSC reset the balance between the two T helper subpopulations, contrasting the prevalence of Th1 over Th2 which is a more protective and immunosuppressive way. In addition, MSC block IL-6 overproduction, a major inflammatory product of monocyte/macrophage cell and effector of acute rejection. Finally, MSC infusion may help prevent HGF abatement in blood and kidney [35].

Beside these paracrine effects, additional studies examined the treatment with the injection of exosomes or MVs from MSC. Exosomes are extracted from BM-MSC and observed under transmission electron microscope (TEM). The expression of surface molecular marker CD63 is positive using flow cytometry. In short, renal outcomes including function parameters and the extent of histological injury were significantly improved by exosomes/MVs in comparison to nontreated controls [36]. MVs have been already tested in AKI model and appear to induce nephroprotection similarly to MSC administration [37]. Interestingly, a number of miRNA species have been shown to play protective roles in ischemic AKI. mir-21 is induced after renal I/R and targets proapoptotic programmed cell death protein 4. This cascade is regarded as one of the main mechanisms involved in delayed preconditioning [38]. Similarly, mir-34a is induced by tumor suppressor p53 and protects against tubular cell injury and death [39]. By contrast, mir-181 seems to be deleterious in I/R injury. Its inhibition leads to the upregulation of Bcl-2 (an antiapoptotic factor) and downregulation of Bax (a proapoptotic factor), thereby causing the protection of proximal tubular cells from injury [40].

On the basis of these encouraging preclinical observations, clinical trials have been launched testing MSC therapy in various settings of renal IRI, including cardiovascular surgery and kidney transplantation (KTx) [41]. In IRI-associated AKI, a large trial included 156 patients undergoing coronary artery bypass grafting. Preliminary results were reported at the meeting of the American Society of Nephrology in November 2014 in Philadelphia, PA. Overall, intra-arterial injection of allogenic human MSC seems to be safe within the 1st year after treatment. In the phase 2 of the treatment, the primary outcome was time to kidney recovery defined as a postoperative serum creatinine return to preoperative baseline values. The first occurrence of a postdosing serum creatinine level that is equal to or less than the subject's preoperative baseline level. The secondary outcome was All-Cause Mortality or Dialysis (composite endpoint). Their first results in phase 2 show that the treatment with allogenic human MSC does not improve the time to complete kidney recovery, mortality, or the need for dialysis (https://clinicaltrials.gov/; NCT01602328).

In living donor-related KTx, the largest study published thus far enrolled 159 patients. The two groups of patients receiving autologous BM-derived MSC were infused either at the time of reperfusion or 2 weeks after KTx. The control group did not receive MSC. After 1 year, the incidence of biopsy-confirmed acute rejection was 7.5 and 7.7%, respectively, in the two MSC-treated groups, whereas it was 21.6% in the control group. Also, kidney function at 1 year was better in the MSC-treated groups, and the patients in these groups presented with less opportunistic infections [42]. Our group has recently analyzed the immunosuppressive effects of MSC administered after KT, as reported at the meeting of the American Transplant Congress in May 2015 in Philadelphia, PA. MSC (1.5–3.0 × 10^6^/kg) infusion was planned 3 to 5 days after KT to 5 patients, who were prospectively screened for anti-HLA antibodies at months 1, 3, and 6. Collectively, there were 23/50 and 29/50 HLA mismatches (MM) with kidney and MSC donor, respectively, out of which 5 were shared MM. We observed that 2 patients developed anti-HLA antibodies against shared kidney/MSC MM and 1 patient developed 2 specific antibodies against MSC (MSCSA) at month 6. All antibodies were anti HLA class I except for 1.

## 5. MSC Therapy in Liver Ischemia/Reperfusion Injury

BM-derived MSC represent a promising candidate for liver cell therapy because of their properties of paracrine signaling, immunomodulation of both adaptive and innate immunity, and possible differentiation into the injured tissue. Kuo et al. have shown that both MSC-derived hepatocytes and MSC, transplanted by either intrasplenic or intravenous way, can be engrafted into the liver and differentiate into functional hepatocytes. Intravenous transplantation was more effective in rescuing liver failure than intrasplenic transplantation [43]. Moreover, they also noticed that MSC were more resistant to ROS* in vitro*, reduced oxidative stress in recipient mice, and accelerated repopulation of hepatocytes after liver damage, suggesting a possible role for paracrine effects.

Some other multiple studies related that autologous adipose tissue-derived MSC (HADMSC) cell administration preserved the integrity of hepatocytes and suppressed inflammatory responses, oxidative stress, and apoptosis in a rodent model of hepatic IRI [44]. HADMSC are effective in decreasing the pathological damage. The inflammatory damage (IL-6) was decreased and the regenerative cells (PCNA-positive cells) increased in group treated with HADMSC.

From these interesting results in experimental animal models, numerous clinical studies have been initiated to investigate the therapeutic potential of MSC. Among 94 registered clinical trials focusing on the utilization of MSC in man, 4 target MSC-based treatments of liver diseases. In general, an average of thirty-two million autologous MSC is administrated through a single injection into the peripheral or portal vein. The treatment is well tolerated and no severe side-effects were observed until the end of the follow-up at 12 months after the transplantation.

All studies concerning MSC into liver failure suggested that autologous MSC infusion allows mild biological improvements in patients, but clear and significant clinical benefit was not reported yet. To our knowledge, none of these studies provided histologic evidence of improvement with MSC treatment. Of note, intraportal infusion seemed to be more efficient than peripheral route [45]. We are currently completing a phase 1-2 study of safety and tolerability in 10 liver transplant recipients under standard immunosuppression receiving 1.5–3 × 10^6^/kg third-party MSC within 3 days after surgery. Primary endpoints are MSC infusion toxicity, incidence of cancer, and opportunistic infections at month 6. Secondary endpoints are patient and graft survival and rejection rates at month 6, as well as the effects of MSC on recipients' immune function and liver histology at month 6 [46].

## 6. MSC Therapy in Cardiac Ischemia/Reperfusion Injury

Cardiac ischemia after an AMI leads to impaired cardiac function and is associated with increased morbidity and mortality. MSC administration in case of cardiac IRI has been associated with a significant reduction of cell death markers and improved viability. Numerous* in vitro* and* in vivo* studies of cardiac IRI have shown the pleiotropic effects of MSC such as antifibrotic, immunomodulatory, antiapoptotic, and proangiogenic features as well as the impact of inflammation/cytokine expression on the different aspects of homing, including chemokine-chemokine receptor interactions, adhesion on endothelial cells, transendothelial migration, and invasion through the extracellular matrix [47]. This protective effect was reproduced by the administration of MSC's conditioned medium alone, suggesting that MSC may exert a paracrine effect. The discovery of VEGF and IGF-1 in the supernatant of MSC in culture correlates with the benefit effect of conditioned medium observed in many studies [48]. In addition, some studies revealed that MVs can be shed from the plasma membrane of MSC and play a role in maintaining cell homeostasis. The injection of MSC-derived MVs in rat improves cardiac function and promotes angiogenesis in ischemic heart by increasing the numbers of blood vessels [49]. Different kinds of miRNA participate to the repair of the myocardial tissue. Serum miR-1 levels strongly correlate with myocardial infarction size and with serum level of creatine, which indicates a correlation between miR-1 levels and the extent of myocardial damage [50]. A study analyzing mir-150 KO mice showed a significantly impaired cardiac function and structure after AMI in comparison to controls. miR-150 KO mice present with higher numbers of TUNEL-positive cells, increased neutrophil infiltration, and increased necrosis and disorganized structure after 1 day of AMI when compared to WT mice [51].

Many studies injected MSC few hours after the reperfusion. In these cases, the deterioration of the endothelial cells and activation of lethal reperfusion injury occur within the first minutes of reflow. This is why some of the research in the fields of cardiac injury injected MSC at the onset of reperfusion. These studies observed reduction of myocardial injury likely related to attenuation of reperfusion injury.

In 2014, Heldman et al. show that mesenchymal adult stromal cells (MASC) exert regenerative and antifibrotic effects within the myocardium and that these effects were associated with improved functional capacity and quality of life. In a repeated measures model, the 6-minute walk test, which measures the distance of a patient able to walk over a total of six minutes on a hard, flat surface, increased in the MSC-treated group but not in the placebo groups. At 6 months, the mean change from baseline in distance walked was 28.2 meters and 21.6 meters in MSC-treated versus control patients, respectively. Ongoing exploration of cell-based therapy for ischemic cardiomyopathy is warranted [52].

## 7. MSC Therapy in Cerebral Ischemia/Reperfusion Injury

Cerebral ischemia is a major cause of morbidity and mortality in the aged population. During cerebral infarction, transplanted MSC migrate to damaged brain tissue and may assume neural phenotypes. They are also able to inhibit apoptosis and to exert neuroprotection by expressing neurotrophic factors in addition to stimulating endogenous factors. The secretion of cytokines by MSC may have immunomodulatory, angiogenic, anti-inflammatory, and antiapoptotic effects and also contributes to the modulation of acute and chronic pathological conditions [53]. MSC have been tested using two routes of administration, that is, intracranially (intrastriatal or intracerebroventricular) or intravascularly (intra-arterial or intravenous). Intrastriatal MSC transplantation at day 1 after stroke significantly increased axonal sprouting and remyelination in the cortical penumbra [54]. Increasing evidence shows that intravascular cell administration after stroke is a viable alternative to intracranial transplantation. Intravascular delivery may be better for larger lesions as it could lead to a wider distribution of transplanted cells around lesions than intracranial delivery [55].

In the early stage of cerebral infarction, MSC have a stimulating effect on the expression of various growth factors in the ischemic zone, namely, brain-derived neurotrophic factor (BDNF), nerve growth factor (NGF), basic fibroblast growth factor (bFGF), IGF, HGF, VEGF, angiogenic factor, and stem cell factor. All of these factors may facilitate functional recovery by inducing angiogenesis, reducing neuronal apoptosis, rebuilding synapses and dendrites, and enhancing axonal regeneration and differentiation of endogenous neural stem [56]. Thus, MSC can upregulate soluble factors, such as bone morphogenetic protein 2 and bone morphogenetic protein 4. These factors are known to play a key role in astrocytic differentiation in ischemic area and improve the level of the gap junctional protein connexin-43 (CX-43), which in turn permits the exchange of small molecules in brain and enhances synaptic efficacy [57].

Another beneficial effect of the injection of MSC in the cerebral stroke is their immunomodulatory effects. Indeed, MSC inhibit the proliferation and the cytotoxicity of T cells and then reduce the production of IFN-*γ*. Thus, IL-10 induces the protective effect of Tregs, which can control the activation of proinflammatory T cells and decrease inflammatory IFN-*γ* [58]. A recent study compares the cerebral stroke treatment with the injection of conditioned medium of baseline rat BM-derived MSC* versus* MSC derived from rat BM after cerebral ischemia. They first demonstrated that there was no impact of cerebral stroke on morphology and cell surface marker expression of BM-MSC. Then they showed that administration of conditioned medium from normal MSC or stroke-MSC does not reduce the extent of brain infarction* in vivo*. However, the authors demonstrated a significant functional neuroprotection by infusion of MSC medium, which supports that MSC derivatives may become a novel therapeutic strategy in ischemic stroke [59].

## 8. Perspectives

IRI represents a worldwide public health issue of increasing incidence, which affects various organs and tissues and is associated with a significant morbi-mortality. In the absence of specific target-oriented therapy, the treatment of patients presenting with IRI mostly relies on supportive maneuvers [60]. The pathophysiology of IRI leads to both immune and metabolic consequences. MSC have demonstrated immunomodulatory, anti-inflammatory, and tissue repair properties in many rodent studies and in ongoing clinical trials. Their administration at the time of IRI and/or at later times may attenuate its severity and accelerate the regeneration process. Even more promising, MSC derivatives have proven efficient in animal models, which further emphasize the role of paracrine mediators in MSC therapy and may help avoid total cell infusion. The testing of MSC therapy in preliminary clinical trial shows encouraging results and opens novel avenues in the management of IRI and SOT.

## Figures and Tables

**Table 1:** Immune impact of mesenchymal stromal cells.

	Cytokines	Sources
Upregulation	IL-6, -10, -11, -12, -13, TGF-*β*, and NO	Anti-inflammatory M2 macrophages
	IL-4	TH2 lymphocytes

Downregulation	IL-2, IFN-*γ*	TH1 lymphocytes
	TNF-*α*, IL-1*β*	Proinflammatory M1 macrophages
